# The combination of SMRT sequencing and Illumina sequencing highlights organ-specific and age-specific expression patterns of miRNAs in Sika Deer

**DOI:** 10.3389/fvets.2022.1042445

**Published:** 2022-11-14

**Authors:** Boyin Jia, Xue Wang, Fuquan Ma, Xin Li, Xintong Han, Linlin Zhang, Jianming Li, Naichao Diao, Kun Shi, Chenxia Ge, Fuhe Yang, Rui Du

**Affiliations:** ^1^College of Animal Medicine, College of Animal Science and Technology, Jilin Agricultural University, Changchun, China; ^2^Laboratory of Production and Product Application of Sika Deer of Jilin Province, Jilin Agricultural University, Changchun, China; ^3^College of Chinese Medicine Materials, Jilin Agricultural University, Changchun, China; ^4^College of Vocational and Technical Education, Changchun Sci-Tech University, Changchun, China; ^5^Institute of Wild Economic Animals and Plants and State Key Laboratory for Molecular Biology of Special Economical Animals, Chinese Academy of Agricultural Sciences, Changchun, China

**Keywords:** Sika Deer, organ-specific, age-specific, miRNA, expression pattern

## Abstract

Due to the lack of high-quality Sika Deer (*Cervus nippon*) transcriptome and sRNAome across multiple organs or development stages, it is impossible to comprehensively analyze the mRNA and miRNA regulatory networks related to growth, development and immunity response. In this study, we used single molecule-real time sequencing (SMRT-seq) and Illumina sequencing methods to generate transcriptome and sRNAome from ten tissues and four age groups of Sika Deer to help us understand molecular characteristics and global miRNA expression profiles. The results showed that a total of 240,846 consensus transcripts were generated with an average length of 2,784 bp. 4,329 Transcription factors (TFs), 109,000 Simple Sequence Repeats (SSRs) and 18,987 Long non-coding RNAs (LncRNAs) were identified. Meanwhile, 306 known miRNAs and 143 novel miRNAs were obtained. A large number of miRNAs showed organ-specific and age-specific differential expression patterns. In particular, we found that the organ-specific miRNAs were enriched in the brain, some of which shared only between the brain and adrenal. These miRNAs were involved in maintaining specific functions within the brain and adrenal. By constructing miRNA96mRNA interaction networks associated with Sika Deer immunity, we found that miRNAs (miR-148a, miR-26a, miR-214, let-7b, etc.) and mRNAs (CD6, TRIM38, C3, CD163, etc.) might play an important role in the immune response of Sika Deer spleen. Together, our study generated an improved transcript annotation for Sika Deer by SMRT-seq and revealed the role of miRNA in regulating the growth, development and immunity response of Sika Deer.

## Introduction

Sika Deer (*Cervus nippon*) is an important economic animal for the purpose to obtain antler and meat. It is widely cultivated in China, Japan and Korea. However, as a non-model animal, the genomic sequence information of Sika Deer is limited, which limits the development of molecular breeding of Sika Deer. In particular, various pathogens have been causing infectious diseases and deaths of Sika Deer. Therefore, there is an urgent need for a long fragment gene sequence that can identify the growth, development and immunity, which will contribute to selective breeding as well as disease prevention and control in Sika Deer.

As a third-generation sequencing method, single molecule-real time sequencing (SMRT-seq) can generate long transcripts without requiring further assembly (1). This method effectively overcomes the problem of the second-generation sequencing that reads length are short and cannot span the entire transcript (2). SMRT technology provided high-quality and complete transcripts by directly reading reverse-transcribed full-length complementary cDNA through the advantage of long read-length (3). SMRT technology is helpful to solve the problems in genome, transcriptome and epigenetic research. This method has been widely used to characterize the full-length transcriptome of animals and plants to collect transcriptome data that reflects the complete species sequence (4–6). As far as we know, the full-length transcriptome sequencing of Sika Deer has not been reported.

In the process of transcription, a large number of non-coding RNAs have regulatory functions (7). MiRNA, as a non-coding RNA of 20–24 nt, cleaves its target or inhibits target translation at the post-transcriptional level (8). It plays a crucial role in gene expression, cell development, immune regulation, tumorigenesis and other life processes (9). Currently, the research on miRNAome of Sika Deer mainly focused on the rapid growth and regeneration of antler, the growth of muscle, the testis development and spermatogenesis (10–12). It lacks the analysis of miRNA expression profiles of different organs and development stages of Sika Deer. MiRNAome studies on other species have shown great organ- and age-dependent variations in miRNA expression. Isakova et al. identified ~400 organ-dependent miRNAs in mice (13); Wang et al. identified 740 organ-dependent miRNAs in giant panda (14); Jie et al. identified 275 organ-dependent miRNAs in Chinese forest musk deer (15); Liu et al. identified 312 age-dependent miRNAs in the citrus red mite (16). In particular, the highly ordered distribution of immune cells in the spleen makes it the largest peripheral immune organ to resist pathogens (17). Many miRNAs are involved in the regulation of immune response (18). However, there are few reports on the regulatory mechanism of miRNAs in the Sika Deer spleen. It is an urgent need to comprehensively examine the spatiotemporal expression changes of miRNAs that regulate the immune response of Sika Deer. In conclusion, we hope to conduct a comprehensive study on miRNAome of different development stages and organs, which will provide new insights into miRNA regulation of growth, development and immunity in Sika Deer.

Here, we provided the complete full-length transcriptome of Sika Deer using RNA-Sequencing (RNA-seq) combined with SMRT-seq. Using the procured transcriptome data, we analyzed coding sequence (CDS), Transcription factors (TFs), Simple Sequence Repeats (SSRs), Long non-coding RNAs (LncRNAs) and gene function annotation. This full-length transcriptome contributed to the comprehensive dataset of genetic information of Sika Deer. In addition, we identified and characterized the miRNA expression profiles from ten tissues of Sika Deer throughout four different life stages using sRNA-seq. We obtained a large number of Sika Deer organ-specific, age-specific miRNAs. Finally, we obtained miRNAs and their target genes related to immunity by constructing the miRNA-mRNA interaction network of the spleen. The above findings will provide a lot of resources for further analysis of the complexity of the growth, development and immunity of Sika Deer.

## Materials and methods

### Animals tissue and RNA extraction

All samples of Sika Deer used were collected from the Institute of Special Economic Animals and Plants of Chinese Academy of Agricultural Sciences. The grouping of 12 Sika Deer was as follows: juvenile (1 year old; *n* = 3), adolescence (3 years old; *n* = 3), adult (5 years old; *n* = 3), and aged (10 years old; *n* = 3). After all Sika Deer were anesthetized and euthanized, ten types of tissue were collected for this study including the adrenal, antler, brain, heart, kidney, lung, liver, skeletal muscle, spleen and testes. Then, the total RNA of each tissue was extracted with TRIzol (Invitrogen, USA). The concentration, purity and integrity of RNA samples were detected by agarose gel electrophoresis, NanoDrop (Nanodrop products, USA) and Agilent 2100 Bioanalyzer (Agilent Technologies, USA).

### PacBio SMRT-seq library construction and sequencing

The total RNA samples from ten tissue types at four development stages of Sika Deer were mixed equally based on their concentrations together for PacBio SMRT-seq library construction. High throughput sequencing was completed at the Novogene Bioinformatics Institute (Novogene, China). Briefly, the SMARTer™ PCR cDNA Synthesis Kit (Clontech, USA) was used to reverse transcribe mRNA into DNA. Next, the BluePippin (Sage Science, USA) was used to perform fragment screening of the full-length cDNA for damage repair, end repair, the connection of SMRT dumbbell joints, and exonuclease digestion. Finally, a total of three fragment size libraries, including 1–2 K (4 SMRT cells), 2–3 K (5 SMRT cells), and 3–6 K (5 SMRT cells), were sequenced on the PacBio platform.

### Illumina RNA-seq and sRNA-seq library construction and sequencing

According to the method we described earlier, 40 cDNA libraries were generated using NEBNext^®^ Ultra™Directional RNA Library Prep Kit for Illumina^®^ (NEB, USA) following manufacturer's recommendations (19). After using TruSeq PE Cluster Kit v3-cBot-HS (Illumia) to generate cluster on cBot Cluster Generation System, the library preparations were sequenced on an Illumina platform.

Forty small RNA libraries were generated from 120 samples of ten tissues at four development stages. Briefly, equal amounts of RNAs from three individuals were pooled to construct small RNA libraries using a TruSeq small RNA Sample Pre Kit for each developmental stage (Illumina, USA). Small RNAs with a length of 18–30 nt were collected, followed by high throughput sequencing based on Illumina Hiseq 2,500 (Illumina Inc., USA). 50 bp single-end reads were generated after the clustering of the index coded samples, performed on a cBot Cluster Generation System using TruSeq SR Cluster Kit v3-cBot-HS. After masking the adaptor sequences and removing reads with excessively small tags or from contaminating adapter-adapter ligation, the clean reads were processed for computational analysis. These filtered clean reads were aligned against with NCBI, Repeat Masker and Rfam, allowing a maximum of rwo mismatches to remove repeating ribosomal RNA (rRNA), small interfer RNA (siRNA), small nucleolar RNA (snoRNA), nuclear small RNA (snRNA), transport RNA (tRNA), other non-coding RNAs. The remaining reads was followed by miRBase 22.0 to compare and identify known miRNAs, and predict new miRNAs with miRdeep software 2.0.5 (20).

### Gene structural analysis and functional annotation

Long reads were analyzed using SMRT Analysis software package to obtain full-length consensus transcripts (21). According to the characteristics of the reads of interest (ROI), it can be divided into full-length non-chimeric reads (FLNC), full-length chimeric reads (FLC), and non-full length reads (NFL). Among them, FLNC reads were clustered using iterative isoform-clustering (ICE). Then, the quiver algorithm was used to subsequently polish FLNC reads to create the final consensus transcriptome. All the isoforms were corrected with Illumina short reads using the software LoRDEC (v0.9) (22). The redundancies in the corrected transcript were then removed using the CD-HIT software with the following parameters: -c 0.95 -T 6 -G 0 -aL 0.00 -aS 0.99 to obtain the final transcript (23). Transcript function was annotated based on the following databases: NR (24), Swissprot (25), KEGG (26), KOG (27), GO (28), NT and Pfam (29) using E-value 10^−5^ as a cutoff. The CNCI (30), Pfam-scan (31), PLEK (32) and CPC (33) tools were used to predict the coding potential of transcripts. SSR of the transcriptome was identified using MISA (http://pgrc.ipkgatersleben.de/misa).

### Comparison of miRNA expression profiles in different organs and development stages

In this study, |log2 (foldchange)|>1 and qvalue<0.01 were used as screening criteria to determine the differential expression level of miRNA by using the DEGseq (2010) R package (34). The target genes of differential expression (DE) miRNA were predicted using miRanda software (35). We mapped all the target genes of DE miRNAs to the terms of GO database for GO enrichment analysis. The KEGG database was used for pathway analysis of target genes, and the pathway entries enriched in target genes were identified. GO analysis and KEGG analysis were performed with the DAVID Bioinformatics Resources v6.7 (http://david.abcc.ncifcrf.gov/). Fisher's exact test was used to define significant GO and KEGG as having *p*-value < 0.05.

### Analysis of development-dependent miRNA expression patterns

The comparison was made between two adjacent development stages of each organ, with the younger development stage as the denominator, namely adolescence vs. juvenile (2 vs. 1), adult vs. adolescence (3 vs. 2), and aged vs. adult (4 vs. 3). The fold change of miRNAs ≥2 was classified as the upregulated pattern (U); the fold change of miRNAs ≤ 0.5 was classified as the downregulated pattern (D), and the remaining miRNA was classified as the maintained pattern (M). In total, 27 combinations were possible.

### Analysis of the miRNA-mRNA interaction network

The miRanda database was used to predict the target mRNAs of DE miRNAs and to intersect with the mRNA sequencing data. Their expression patterns were evaluated using the Pearson correlation coefficient. When the expression of target mRNA was negatively correlated with the miRNA, this mRNA could be used as a candidate target mRNA. Finally, the candidate target mRNAs were visually analyzed by Cytoscape 3.5.1 (36).

### Data availability

The sequencing data has been submitted to the NCBI Gene Expression Omnibus. The accession number was GSE212478.

## Results

### Combined sequencing of Sika Deer transcripts

**Figure 1:**
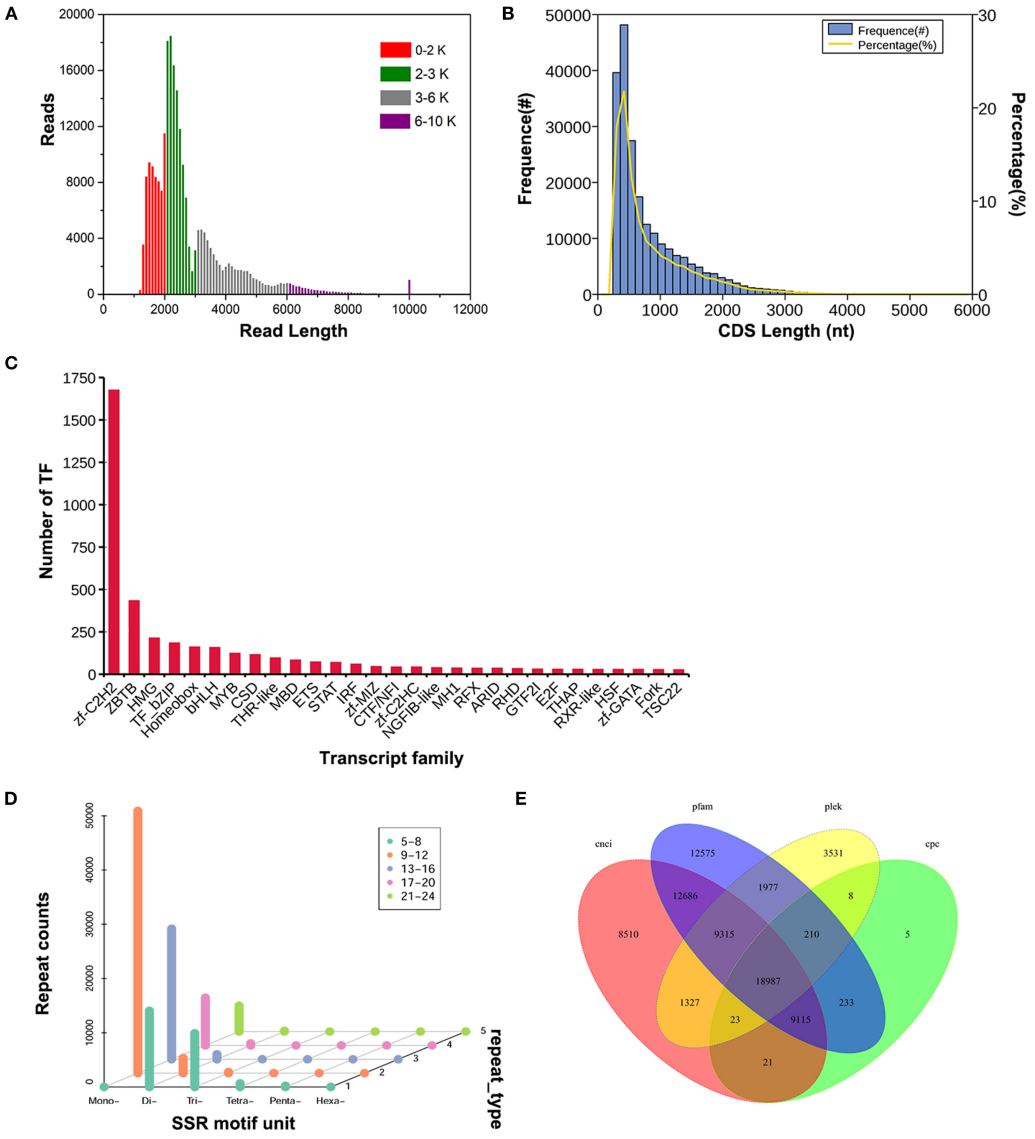
Structure analysis of Sika Deer full-length transcriptome data. **(A)** Length distribution of Sika Deer reads in four fragment size ranges. **(B)** Number, percentage and length distributions of coding sequences of transcripts. **(C)** Transcription factor statistic. **(D)** Scattergram of SSR of transcripts. **(E)** Venn diagram of the number of lncRNAs predicted by CNCI, Pfam, PLEK, and CPC protein structure domain analysis.

In this study, we established three fragment size libraries (1–2 k, 2–3 K, and 3–6 k) were sequenced on the PacBio platform. A total of 19.43 G of raw data and 19.13 G of clean data were generated by 14 SMRT cells (Supplementary Tables 1, 2). There were 7,586,908 reads of insert, including 425,030 full length non-chimeric reads. 2,40,846 common isoforms were obtained, which were divided into four fragment size ranges of 0–2 k, 2–3 K, 3–6 k, and 6–10 k, with the average lengths of 1,681 bp, 2,400 bp, 4,042 bp, and 7,431 bp, respectively (Figure 1A). We further corrected all the consensus isoforms using the previous Illumina read data of 40 mRNA samples from the ten tissues in the four age groups of Sika Deer (19). A total of 148,362 unigenes were obtained, which could be used for subsequent analysis.

### Analysis of CDS, TFs, SSRs, and LncRNAs

CDS prediction was performed on Sika Deer transcripts, and the main distribution range of CDs length was 0–2,000 nt (Figure 1B). TF, as the current research focus, was an important factor affecting the growth and development of animals. We identified 4,329 TFs from 64 families. Among them, the zf-C2H2 (1,676, 38.72%), ZBTB (434, 10.03%), HMG (214, 4.94%), TF_bZIP (185, 4.27%) and Homeobox (161, 3.72%) were the top five transcription factor families (Figure 1C). After analyzing the sequence structure of the transcripts, 109,000 SSRs were detected in 6 nucleotide types. The largest number of single nucleotide repeats and dinucleotide repeats were 37,073 and 10,007, respectively (Figure 1D). lncRNAs were predicted by four programs CNCI, Pfam, PLEK and CPC, with 59,984, 28,602, 65,098, and 35,378, respectively. 18,987 lncRNAs with high confidence were identified (Figure 1E). These results were helpful to understand how genes were expressed and regulated.

### Gene function annotation and categorization

For acquiring a comprehensive annotation of Sika Deer transcriptome, sequences were assigned to align with NR, Swissprot, KEGG, KOG, GO, NT and Pfam, and a total of 131,634, 119,623, 125,888, 88,866, 74,053, 147,893 and 74,053 transcripts were annotated, respectively (Supplementary Figure 1A). 148,065 transcripts were annotated in all databases. 74,053 annotated transcripts could be divided into 56 GO terms (Supplementary Figure 1B). In biological processes, cellular process was the most abundant annotation. In cellular component, the main proportion referred to cell part, cell and organelle. In molecular functions, the genes involved in binding was the most abundant term. In the KEGG database, transcripts were annotated with 46 sub pathways from 6 pathways (Supplementary Figure 1C). Briefly, there were 41,759 unigenes involved in Human Diseases related pathways, in which cancers: overview (7,687 unigenes), Infectious disease: viral (5,240 unigenes) and Endocrine and metabolic diseases (5,170 unigenes) were the top three pathways. There were 24,710 unigenes involved in Metabolism related pathways, in which 3,976 unigenes were predicted to Lipid metabolism, 3,970 unigenes were predicted to Carbohydrate metabolism, and 3,556 unigenes were predicted to Amino acid metabolism. In addition, there were 34,066 unigenes involved in the Organismal Systems related pathways. The most abundant pathways were Immune system (10,306 unigenes) and Endocrine system (6,714 unigenes).

### Overview of small RNA library

We performed small RNA sequencing from ten tissues at four development stages batched to identify a Sika Deer miRNAs expression profile. An average of 12,277,285 raw reads (from 10,518,583 to 15,775,844) and 11,945,167 clean reads (from 10,299,549 to 15,528,146) was obtained using 40 libraries (Supplementary Table 3). These sequences were further mapped to the bovine reference genome (Supplementary Table 4). After comparing the clean reads with the Rfam database, 3.4% of clean reads were annotated to miRNA, rRNA, tRNA, snRNA, and snoRNA (Supplementary Table 5). In addition, comparing the size distribution of miRNA sequences in different organs and development stages, two clear peaks were shown at 20–24 nts and 30–33 nts (Supplementary Figure 2). We discovered 306 known miRNAs and 143 novel miRNAs from ten organs at four development stages of Sika Deer (Supplementary Table 6). These known miRNAs belonged to 164 conserved families and 34 non-conserved families (Supplementary Table 7). Several conserved miRNA families had extremely high expression levels, such as miR-1 (27,948,543 reads), let-7 (83,75,515 reads), miR-26 (30,73,218 reads), miR-378 (20,94,4871 reads) and miR-199 (16,81,847 reads). In contrast, the number of reads of non-conserved miRNA families was relatively lower. For example, the total read counts of miR-2403, miR-2447, miR-2424 and miR-6528 families in all 40 libraries were <100.

### miRNA expression patterns in different organs and development stages

**Figure 2:**
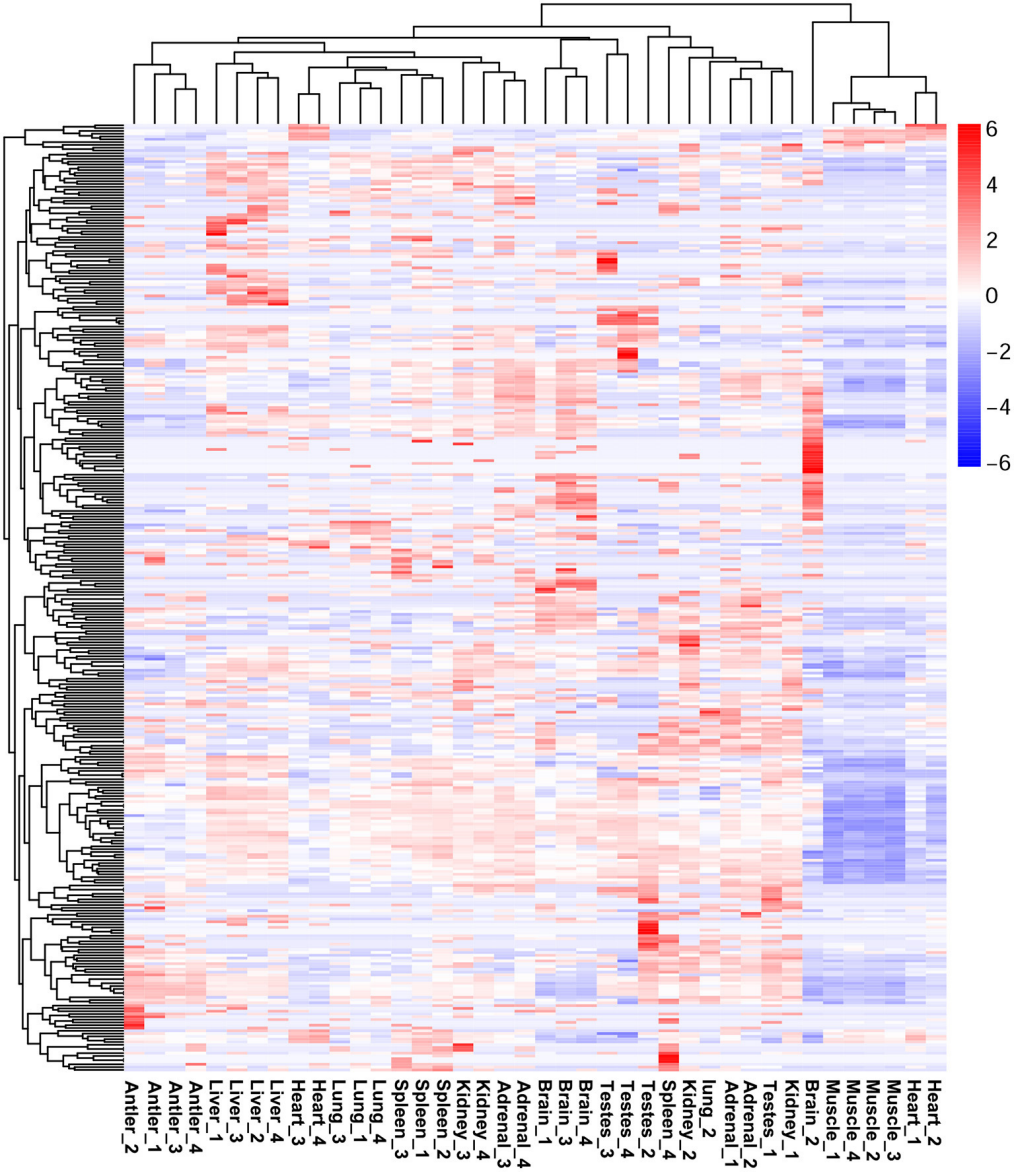
Heat map showing DE miRNAs expression profiles.

The expression profiles of miRNAs in different organs of Sika Deer were significantly different (Supplementary Figure 3). Among them, the number of miRNAs was the largest in the adrenal (314) and spleen (308); the number of miRNAs was the least in muscle (231) and antler (242) (Supplementary Table 7). Sixty six miRNAs were ubiquitously expressed in ten tissues at four development stages. In particular, many miRNAs were constantly highly expressed at all developmental stages, such as let-7b, miR-7, miR-26a, miR-26c, miR-30c, miR-145,miR-199a-3p, miR-409b, miR-3596, and miR-3604. In order to understand the specific expression of miRNAs, we performed cluster analysis on the miRNA expression patterns of ten organs at four development stages (Figure 2). The results showed that miRNAs in the antler, liver and skeletal muscle mainly showed organ specificity. However, miRNAs in the adrenal, brain, heart, kidney, lung, spleen and testes mainly showed development stage specificity. In particular, miRNA expression of the heart, kidney and adrenal in the juvenile and adolescence differed significantly from that of adult and aged; miRNA expression of testis in the juvenile differed significantly from that of adolescence, adult and aged; miRNA expression of spleen in the aged differed significantly from that of juvenile, adolescence and adult. This result reflected the pre- and post-maturity difference.

### Comparisons of miRNAs expression patterns of different organs

**Figure 3:**
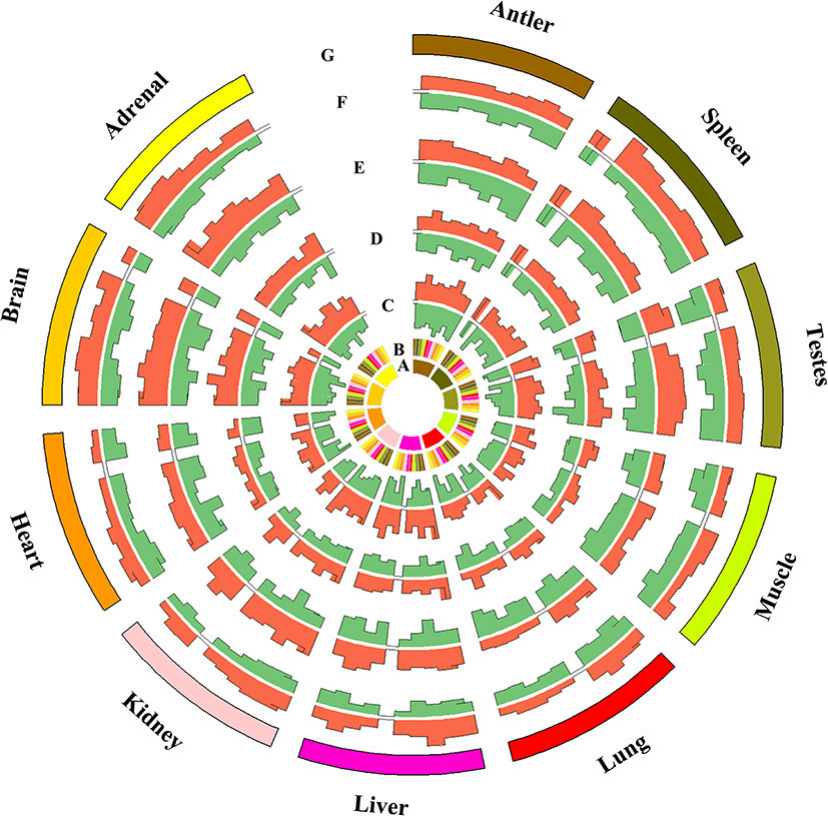
Comparison of miRNAs differentially expressed between any two tissues at the same stage (fold change ≥2 or ≤ 0.5, q value ≤ 0.005).

We compared the different expression levels of miRNAs in any two tissues (*q*-value < 0.01 and |log2 (foldchange)|>1) (Figure 3). The different expression patterns of miRNAs of Sika Deer in all organs at all life stages were examined. For overexpressed miRNAs, the brain had the largest number, followed by the testes, adrenal, kidney, spleen, liver, antler, lung, heart and muscle. For underexpressed miRNAs, the testes had the largest number, followed by the brain, antler, spleen, lung, heart, liver, kidney, adrenal and muscle. We identified organ-enriched miRNAs, which were specific and highly expressed to each organ (Table 1, Supplementary Table 8). When a 2-fold change was used, we identified 234 organ-enriched miRNAs. 47 (20.09%) miRNAs were detected specifically in the brain, 41 (17.52%) miRNAs in the liver and 35 (14.96%) miRNAs in the testes. Meanwhile, we obtained many organ-specific miRNAs, such as miR-219, miR-488 and miR-760-5p in the brain, miR-767 in the heart, miR-2428 in the liver and miR-545-3p in the testis. We also observed that multiple miRNAs existed in some organs, but absented in others, such as miR-329a, miR-496, miR-153, miR-383 and miR-212, which were shared only between the brain and adrenal.

**Table 1:** The number of organ-enriched miRNAs based on fold change.

**Fold change**	**Adrenal**	**Antler**	**Brain**	**Heart**	**Kidney**	**Lung**	**Liver**	**Muscle**	**Spleen**	**Testes**	**Total**
2	26	14	47	16	27	6	41	5	17	35	234
4	18	4	27	12	11	4	22	5	10	24	137
8	12	3	18	10	7	4	19	5	10	18	106
16	11	2	14	10	4	4	15	5	7	12	84
32	11	1	14	9	3	4	15	5	6	10	78
64	11	1	13	9	3	4	13	5	4	8	71
128	11	1	12	9	3	4	13	5	4	8	70
256	11	1	11	9	3	4	13	5	4	8	69

**Figure 4:**
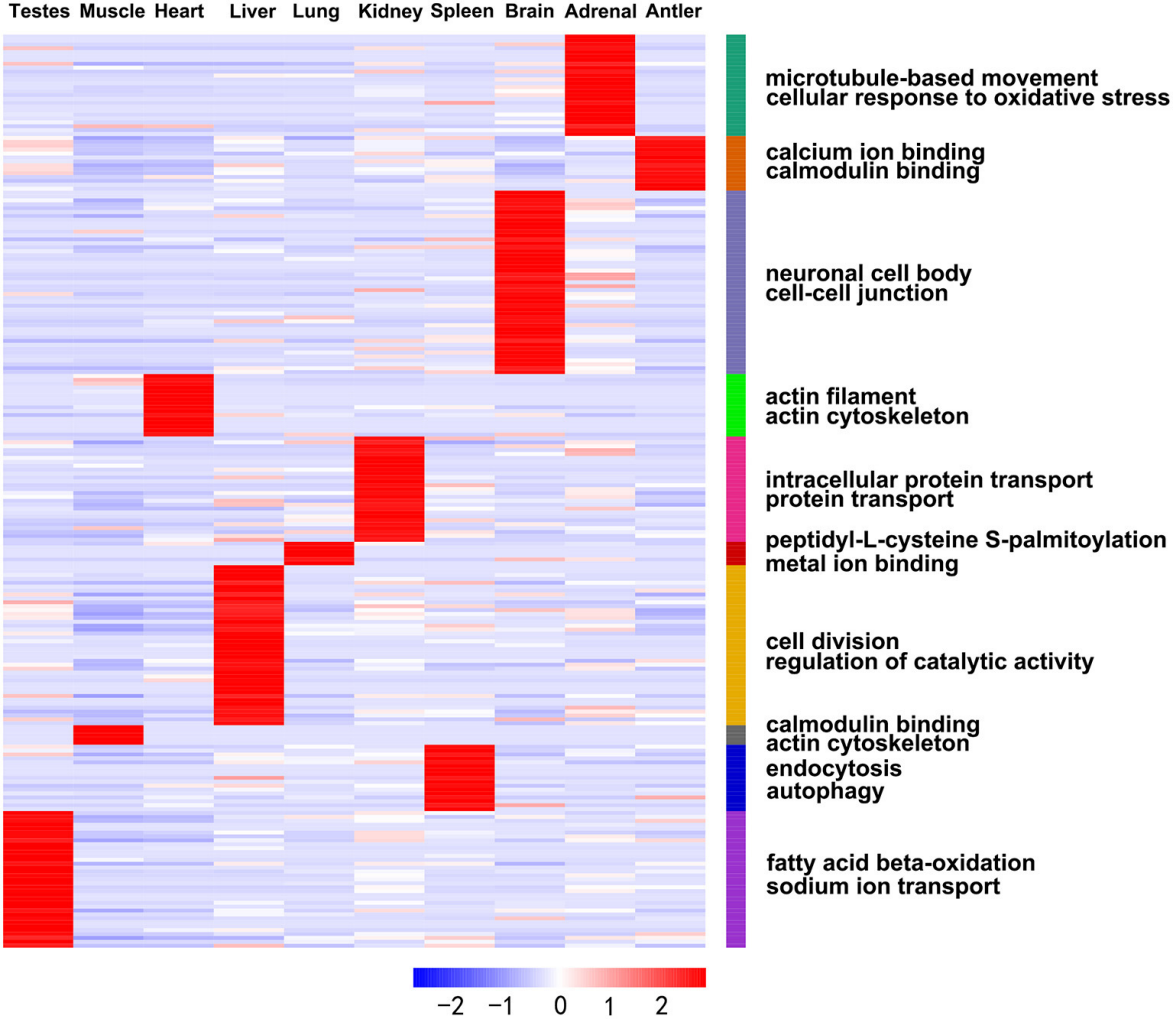
Heat map showing expression profiles of the organ-enriched miRNAs and their target genes with significant GO annotations using the DAVID functional annotation tools.

In addition, we obtained DE miRNA-target genes in ten tissues of Sika Deer annotated in the Swiss-Prot database. GO analysis showed that the ranking of GO terms of organ-enriched miRNAs was associated with biological functions (Figure 4, Supplementary Table 9). For example, the target genes of antler-enriched miRNAs were related to calcium ion binding and calmodulin binding; the target genes of spleen-enriched miRNAs were related to endocytosis and autophagy. Similarly, KEGG analysis showed that the pathways of organ-enriched miRNAs were associated with biologic processes in this study. For example, the target genes of muscle-enriched miRNAs were related to regulation of actin cytoskeleton, the glycolysis/gluconeogenesis; the target genes of spleen-enriched miRNAs were associated with complement and coagulation cascades, platelet activation (Table 2).

**Table 2:** Characteristic pathways and biological processes of the organs as demonstrated by the organ-enriched target genes of DE miRNAs.

**Tissue**	**Characteristic pathways and processes**
Adrenal	Metabolic pathways, Thyroid hormone signaling pathway, Insulin resistance
Antler	HIF-1 signaling pathway, PPAR signaling pathway, Dopaminergic synapse
Brain	PI3K-Akt signaling pathway, MAPK signaling pathway, GnRH signaling pathway
Heart	Platelet activation, Arrhythmogenic right ventricular cardiomyopathy, Focal adhesion
Kidney	Arginine and proline metabolism, Fatty acid metabolism, Cysteine and methionine metabolism, Glyoxylate and dicarboxylate metabolism, Tryptophan metabolism
Liver	Biosynthesis of amino acids, Arginine biosynthesis, N-Glycan biosynthesis, beta-Alanine metabolism, 2-Oxocarboxylic acid metabolism
Lung	Autophagy – animal, Peroxisome
Muscle	Glycolysis / Gluconeogenesis, Regulation of actin cytoskeleton
Spleen	Complement and coagulation cascades, Platelet activation
Testes	Progesterone-mediated oocyte maturation, Oocyte meiosis, Prostate cancer

### Comparisons of miRNAs expression patterns of different development stages

**Figure 5:**
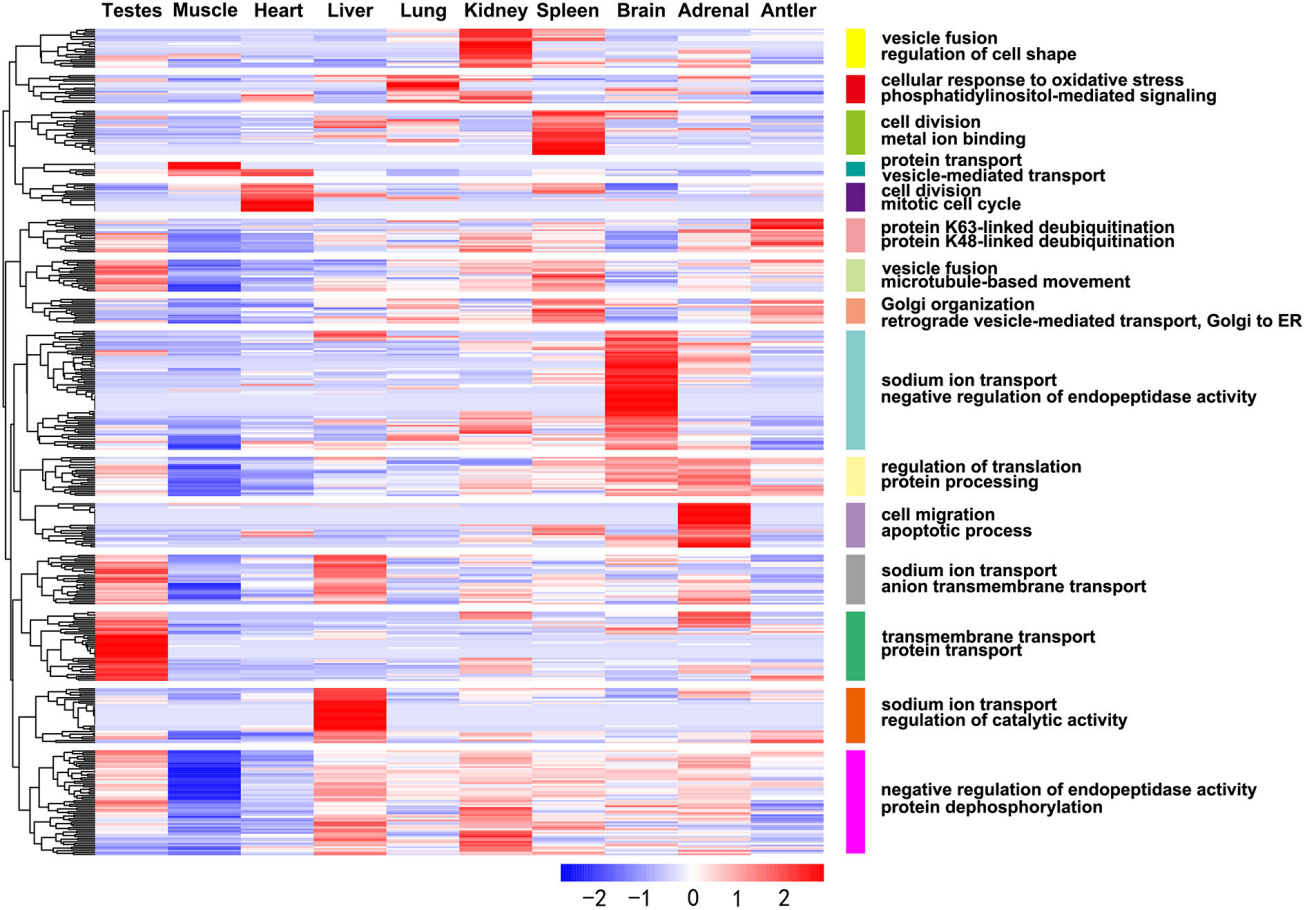
Heat map showing expression profiles of the development-dependent miRNAs and their target genes with significant GO annotations using the DAVID functional annotation tools.

The development-dependent miRNA expression at four development stages was examined based on the *q*-value < 0.01 and |log2 (foldchange)|>1 (Table 3). The results showed that the number of development-dependent miRNAs varied by tissue, ranging from 705 in the testes to 56 in the muscle. We found that development-dependent miRNAs were mainly enriched in the juvenile vs. aged group, the adolescent vs. aged group, and the adolescent vs. adult group. Among them, the DE miRNA of spleen, liver, adrenal, antler and muscle were the most significant in the juvenile vs. aged group; the DE miRNA of testis, brain, kidney and heart was the most significant between adolescence and other stages (adults and aged). The target gene of development-dependent miRNAs were annotated in the SwissProt database. GO analysis showed that the ranking of GO terms of development-dependent miRNAs was usually related to cellular response to oxidative stress, cell division, cell migration, protein transport, protein processing, sodium ion transport, transmembrane transport, microtubule-based movement, apoptotic process and regulation of catalytic activity (Figure 5, Supplementary Table 10).

**Table 3:** Number of DE miRNAs in two different developmental stages.

**Organ**	**1 vs. 2**	**1 vs. 3**	**1 vs. 4**	**2 vs. 3**	**2 vs. 4**	**3 vs. 4**	**Total**
Adrenal	35	75	102	93	68	31	404
Antler	36	38	73	46	73	23	289
Brain	116	71	68	111	133	24	523
Heart	48	35	38	53	65	28	267
Kidney	50	80	76	104	85	43	438
Liver	57	45	67	38	41	35	283
Lung	92	37	48	75	85	21	358
Muscle	11	15	17	4	8	1	56
Spleen	23	33	120	30	92	110	408
Testes	108	129	114	141	130	83	705

**Figure 6:**
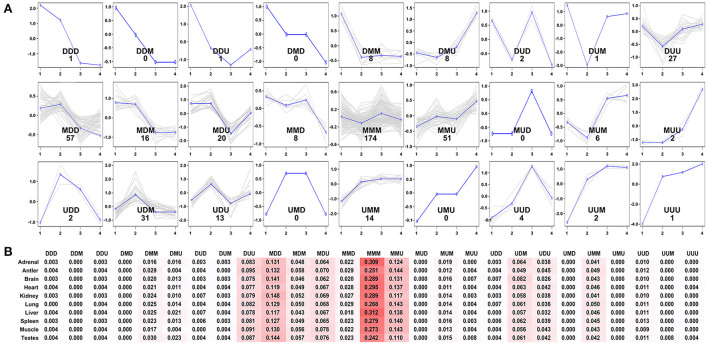
Expression patterns of the development-dependent miRNAs of the Sika Deer. **(A)** The 27 expression patterns of the DE miRNAs obtained by ANOVA between any two sequential stages of development. **(B)** The percentages of miRNAs expression patterns in each tissue.

To further evaluate the expression pattern of development-dependent miRNAs, we compared the miRNA expression levels of any two adjacent development stages by using the younger development stage group as the denominator (Figure 6). Twenty seven expression patterns were determined. Simply, “U” (up) represented the increase across all boundaries; “M” (maintain) represented the similar across all boundaries; “D” (down) represented the decrease across all boundaries. The results showed that most miRNA expressions did not change significantly during aging (MMM: 174 miRNAs), only a few miRNAs showed continuous increase (UUU:1 miRNA) or decrease (DDD:1 miRNA). Then, the beginning of adolescence and adult leaded to significant changes in miRNA (MDD: 56 miRNAs; MMU: 51 miRNAs), which indicated that these two-time nodes were critical in the whole life cycle of Sika Deer.

### Identification of miRNA-mRNA regulatory network associated with the immune response

**Figure 7:**
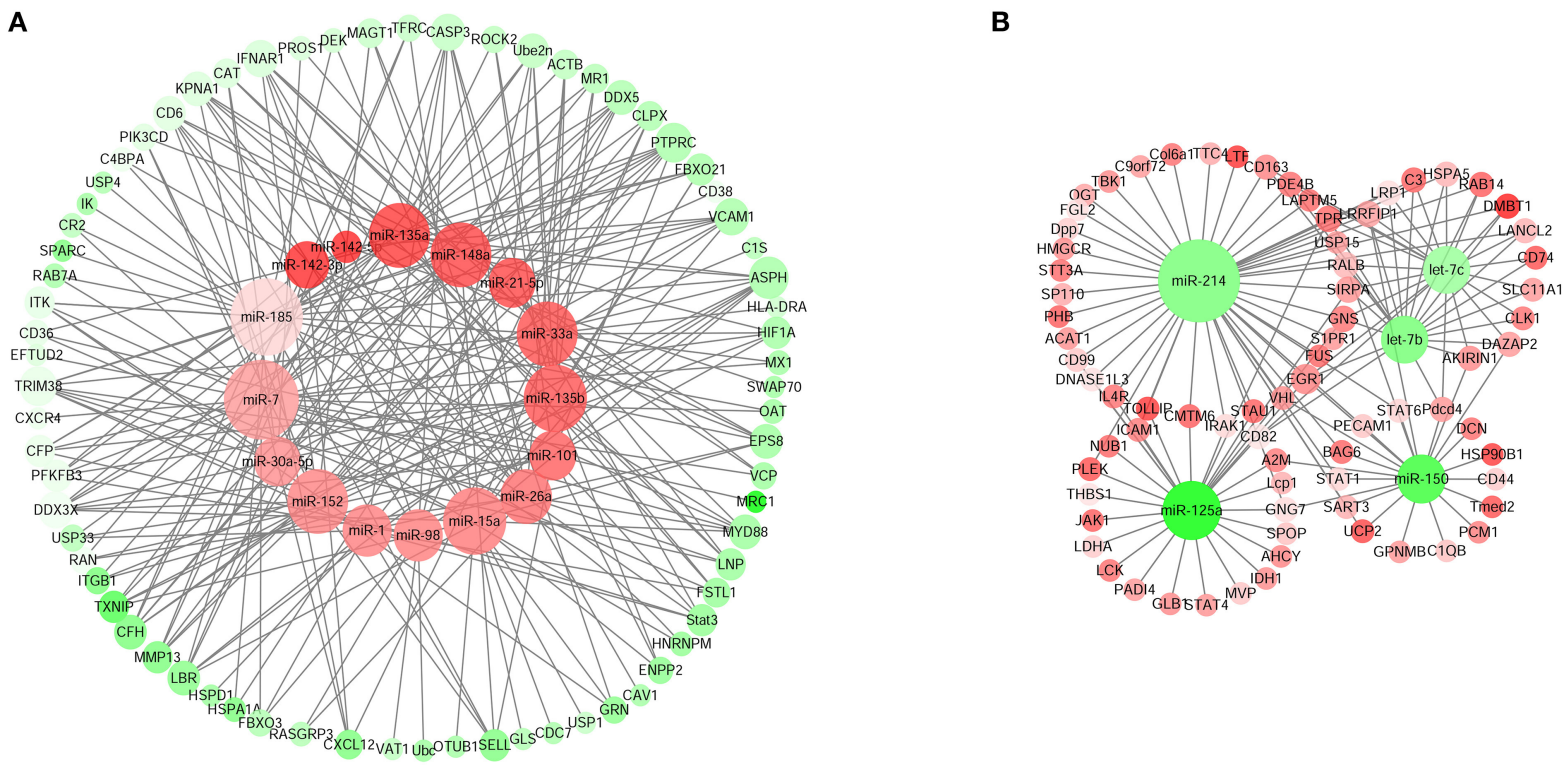
MiRNA–mRNA interaction networks related to immune response in spleen. **(A)** Up-regulated miRNAs and down-regulated target genes related to immune response in spleen. **(B)** Down-regulated miRNAs and up-regulated target genes related to immune response in spleen. The up-regulated miRNAs or genes were displayed as red circles, and the down-regulated miRNAs or genes were displayed as green circles.

We integrated the miRNA-mRNA regulatory network associated with the immune response of the Sika Deer spleen. The results showed that 21 miRNAs (393 pairs of miRNA-mRNA) were negatively associated with the immune response genes (Figure 7). Figure 7A showed that miR-1, miR-7, miR-15a, miR-21-5p, miR-26a, miR-30a-5p, miR-33a, miR-98, miR-101, miR-135a, miR-135b, miR-142-3p, miR-142-5p, miR-148a, miR-152, and miR-185 were up-regulated which targeted STAT3, MyD88, CD6, TRIM38, ITK, etc. related to immune response. In contrast, Figure 7B showed that miR-125a, miR-150, let-7b, miR-214, and let-7c were down-regulated which targeted C3, CD163, EGR1, DMBT1, etc. related to immune response. We speculated that these miRNAs and their target mRNAs might play an important role in regulating the immune response of Sika Deer.

## Discussion

The third-generation sequencing had significantly improved the *de novo* assembly of the transcriptome in species lacking reference genomes (37). Although the single base reading error rate of the third-generation sequence technology was quite high, which could be corrected by combining short and long-read sequencing technologies (38). In this study, we collected RNA samples from ten organs at four development stages of Sika Deer. We obtained 7,586,908 complete transcripts of Sika Deer using SMRT-seq combined with RNA-seq. These complete transcripts were much longer than those obtained by RNA-seq (average length 590 bp and N50 length 787 bp) (19). Among them, a total of 148,065 transcripts of Sika Deer were annotated by homologous genes of seven databases. This provided a reference for classifying new transcripts to obtain gene function information. In mammals, TFs that encode less than one tenth of the whole genome strictly controlled the expression of more than 20,000 genes through complex regulatory networks (39). TFs were important regulatory factors in cell differentiation, tumorigenesis and so on (40). In our study, 4,329 TFs of Sika Deer were identified, which was far more than that of cattle. In the comparison of the top five transcription factor families, the HMG and Homeobox of Sika Deer were significantly enriched than that of cattle (41). The identification of these TFs will be helpful for the study of growth, development and immunity in Sika Deer.

Besides, lncRNAs were important factors that regulate gene expression and multiple biological processes. Thousand eight hundred and thirty five lncRNAs of antler have been identified by RNA-seq, but the number of lncRNAs obtained by a single tissue was very limited (42). In our study, 18,987 lncRNAs were identified. These newly identified lncRNAs could be used as candidate genes to characterize their functions. It was the first time to perform the full-length transcriptome of Sika Deer. It provided a reference for accurate gene annotation in subsequent studies.

MiRNAs play an important role in gene regulation in animals. However, researchers only focused on the miRNA expression of antler, muscle and testis of Sika Deer (10–12). But the miRNA study of the single organ cannot fully understand the panorama of its regulation of Cervidae growth and development. In view of this, we obtained 306 known miRNAs and 143 novel miRNAs using sRNA-seq from ten tissue types at four development stages of Sika Deer. These known miRNAs belonged to 164 conserved families and 34 non-conserved families. Some conserved miRNA families, including miR-1, let-7, miR-26, miR-378, and miR-199, showed relatively high reads (more than 1,000,000), while the number of reads of non-conserved miRNA families was relatively lower (<100). This was consistent with the previous research that the expression abundance of conserved miRNAs was higher than that of non-conserved miRNAs (43). Furthermore, the size distribution of miRNA sequences showed two clear peaks at 20–24 nts and 30–33 nts. Among them, the peak of 30–33 nts corresponded to piRNA-like small RNAs, indicating that it might play a role in maintaining genome stability during development. The comprehensive analysis of microRNAome undoubtedly provides an important reference for understanding the regulation of growth, development and immunity of Sika Deer by miRNAs.

The analysis of miRNAs expression levels showed that 66 miRNAs were ubiquitously expressed in ten tissues at four development stages. These miRNAs play basic biological functions. However, more miRNAs showed organ-specificity and age-specificity. In our study, the brain, liver and testis had the largest number of organ-enriched miRNAs, while antlers, lungs and muscles had the least. Similar studies, for example, the number of organ-enriched miRNAs in the brain and muscle of mice was the largest, while the lung, liver and spleen were just the opposite (13); the number of organ-enriched miRNAs in the organs of Chinese forest musk deer from more to less was the heart, spleen, liver, muscle, kidney and lung (15). It can be seen that the organ-enriched miRNAs of Sika Deer were significantly different from that of mice, but similar to that of Chinese forest musk deer. Although they were all mammals, there were significant differences in organ-enriched miRNAs between ruminants and rodents. In addition, we identified brain-specific miRNAs, including well-described miR-219 and previously unknown miR-488 and miR-760-5p (44). There were other unknown organ-specific miRNAs, such as miR-767 in the heart, miR-2428 in the liver, and miR-545-3p in the testis. Surprisingly, we also found that some miRNAs, such as miR-329a, miR-496, miR-153, miR-383, and miR-212, were shared only between the brain and adrenal. The results suggested that some miRNAs were involved in maintaining specific functions within the brain and adrenal, such as the hypothalamus-pituitary-adrenal axis. In order to further study the functional correlation of organ-enriched miRNA, we performed GO analysis on their target genes. We found that the enriched GO terms were in line with the biological function of their organs.

We performed cluster analysis of the miRNA expression profile of all tissues at all development stages. The results showed that miRNAs in many tissues except antlers, liver and muscle had obvious development-specificity. In the heart, kidney and adrenal, miRNA expression in the juvenile and adolescent tissues was significantly different from that of adult and aged. This finding might suggest that miRNA was involved in regulating the adolescent-adult transition of Sika Deer tissues (heart, kidney and adrenal) to promote body maturation. MiRNAs expression in juvenile testis was significantly different from that of other development periods. It was basically consistent with the previous view of Sika Deer transcriptome analysis. The results suggested that sexual maturation in adolescence required new miRNAs in the testis to regulate genes expression. The juvenile-adolescent transition of Sika Deer was a key period of the development of gonads, which was particularly important for the future study of its reproduction. In contrast, the spleen had the most significant difference in miRNAs expression between the aged and other development periods. Therefore, it was speculated that miRNAs play a crucial role in maintaining immune homeostasis. We further analyzed 27 possible miRNA expression patterns across the life cycle of the Sika Deer. The results showed that the vast majority of miRNAs remained unchanged in all tissues. However, the beginning of adolescence and adult leaded to significant changes in miRNA. It could be seen that the changes in miRNA expression patterns were not random. Especially from adolescence to adult, miRNAs were involved in regulating the maturation of all organs of Sika Deer.

The spleen was an important organ for the stability of the immune system, which was regulated by many factors (45, 46). Among them, miRNA was an important immune regulatory factor, which participated in the process of disease occurrence and development (47). For the first time, we integrated the regulatory network of miRNAs-mRNAs in the spleen of Sika Deer, thereby identifying the target genes of immune-related miRNAs. In Figure 7A, miR-1, miR-7, miR-15a, miR-21-5p, miR-26a, miR-30a-5p, miR-33a, miR-98, miR-101, miR-135a, miR-135b, miR-142-3p, miR-142-5p, miR-148a, miR-152, miR-185 related to immune response were up-regulated. The target genes of the above miRNAs had multiple central nodes, such as STAT3, MyD88, CD6, TRIM38, ITK, etc. STAT3 was a member of the STAT family, which exhibited important immunoregulatory effects in distinct T-cell and myeloid cell compartments (48, 49). For example, STAT3 achieved immune escape through negative feedback regulation of MHC class I (50). MyD88 was an adaptor protein of multiple signaling pathways, which could mediate TLRs to cause inflammatory response (51). The alternative splice of MyD88 had two subtypes of MyD88L and MyD88S: MyD88L, through LTLR/MyD88/NF-κB signaling pathway, became the core participant of innate immunity; MyD88S promoted pro-inflammatory M1 macrophage polarization through interaction with TLR4 (52). As a costimulatory molecule, CD6 participated in thymus development, T cell activation and immune response (53). Kureel et al. found that the expression level of CD6 in the elderly was significantly lower than that of the young counterparts (54). Our result was consistent with this. TRIM38 was a TRIM family member containing a typical E3 ubiquitin ligase domain. TRIM38 had been shown to play a role of negative feedback regulation through different mechanisms in various innate immune responses and inflammatory pathways (55). ITK, as a Tec family kinase, was mainly expressed in T cells (56). It was the key mediator of the strength of signal delivered by the TCR, regulating processes like T cell development and Th1, Th2, Th9, Th17 responses, etc. (57). In this study, with the aging of the Sika Deer spleen, the up-regulation of immune-related miRNAs inhibited the expression of STAT3, MyD88, CD6, TRIM38, ITK, etc., thereby regulating the immune response.

In Figure 7B, let-7b, let-7c, miR-125a, miR-214 and miR-150 related to immune response were down-regulated. The target genes of the above miRNAs had multiple central nodes, such as C3, CD163, EGR1, DMBT1, etc. C3 was the core component of the complement system and the most abundant complement in serum. C3 was the intersection of complement activation pathways (classical, alternative, and lectin pathway) (58). C3 could increase cell-cell interactions, induce intracellular signals after binding to its receptors, and increase intracellular antigen processing. This contributed to the immune response and immune memory (59). CD163, a class I membrane protein, was used as the most specific marker for monocytes and macrophages (60). Human peripheral blood macrophages showed high CD163 expression in the stage of inflammation regression, while monocytes were just the opposite (60). CD163 played an anti-inflammatory and antioxidant role by regulating the expression of inflammatory factors, which was related to the function of scavengers (61). EGR1 was an immune response related transcription factor (62). EGR1 could induce T cell activation and response (63). EGR1 was also a signaling intermediate in B-cell antigen receptor stimulated B cell functional response (64). DMBT1 was a member of the SRCR superfamily and also a tumor suppressor, playing a role in innate immune defense and inflammation (65, 66). Taken together, it was speculated that the down-regulation of let-7b, let-7c and miR-214 promoted the expression of C3, CD163, EGR1, DMBT1 during the aging process of the spleen, thus regulating immune defense.

## Conclusion

We have successfully provided the full-length transcriptome of Sika Deer by combining SMRT-seq and RNA-seq technologies. The analyses of CDs prediction, TFs prediction, SSRs discovery, LncRNAs prediction and gene annotation were smoothly conducted. This was a major advance in Sika Deer genetics. We identified 306 known miRNAs and 143 novel miRNAs from ten organs at four developmental stages. Many miRNAs displayed organ-specificity and age-specificity.

In addition, we obtained an immune-related miRNA-mRNA interaction network of the Sika Deer spleen. In conclusion, this study provided lines for understanding the roles of miRNAs in the major physiological processes, such as growth, development and immunity in Sika Deer. It also provides important and valuable basis resources for advanced genomic research on Sika Deer.

## Data availability statement

The datasets presented in this study can be found in online repositories. The names of the repository/repositories and accession number(s) can be found in the article/Supplementary material.

## Ethics statement

The animal study was reviewed and approved by the Jilin Agricultural University Committee on the use of live animals. Written informed consent was obtained from the owners for the participation of their animals in this study.

## Author contributions

BJ, RD, and FY: conceptualization. BJ, XW, FM, XL, and XH: methodology. BJ, LZ, JL, and ND: software. BJ: writing—original draft preparation. BJ, KS, and CG: writing—reviewing and editing. RD: supervision. All authors contributed to the article and approved the submitted version.

## Funding

This study was funded by National Natural Science Foundation of China (Grant/Award Number: 32002171), Science and Technology Research Project of Jilin Province Education Department (Grant/Award Number: JJKH20220364KJ), and Jilin Province Major Science and Technology Special Project (Grant/Award Numbers: 20220304001YY and 20220304003YY).

## Conflict of interest

The authors declare that the research was conducted in the absence of any commercial or financial relationships that could be construed as a potential conflict of interest.

## Publisher's note

All claims expressed in this article are solely those of the authors and do not necessarily represent those of their affiliated organizations, or those of the publisher, the editors and the reviewers. Any product that may be evaluated in this article, or claim that may be made by its manufacturer, is not guaranteed or endorsed by the publisher.

## Supplementary material

Supplementary Figure 1 Annotation of transcripts from Sika Deer. **(A)** Gene function annotation in 7 databases. **(B)** GO classification diagram of transcripts. **(C)** KEGG Pathway classification diagram of transcripts.

Supplementary Figure 2 Small RNA length distribution in the libraries. **(A)** The length distribution of unique small RNAs in the four developmental stages of Sika Deer. **(B)** The length distribution of unique small RNAs in ten organs of Sika Deer.

Supplementary Figure 3 miRNA expression in ten organs.
