# Supplementary figures and images for: The combination of SMRT sequencing and Illumina sequencing highlights organ-specific and age-specific expression patterns of miRNAs in Sika Deer
Source: Front Vet Sci. 2022 Nov 14;9:1042445. doi: 10.3389/fvets.2022.1042445 (PMC9701854; doi:10.3389/fvets.2022.1042445)

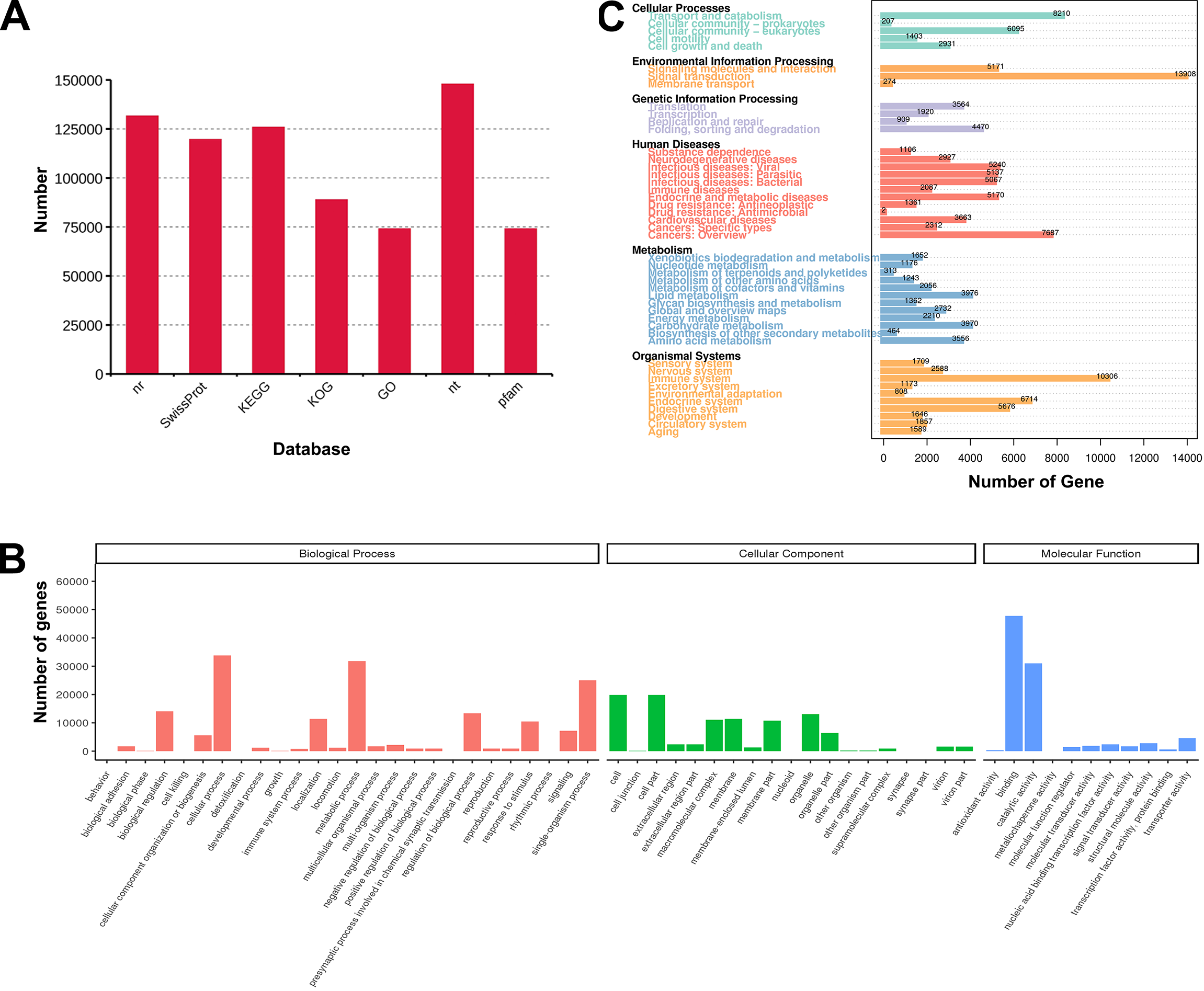

Supplement: Supplementary Figure 1 — Annotation of transcripts from Sika Deer. (A) Gene function annotation in 7 databases. (B) GO classification diagram of transcripts. (C) KEGG Pathway classification diagram of transcripts. [file Image_1.TIF]

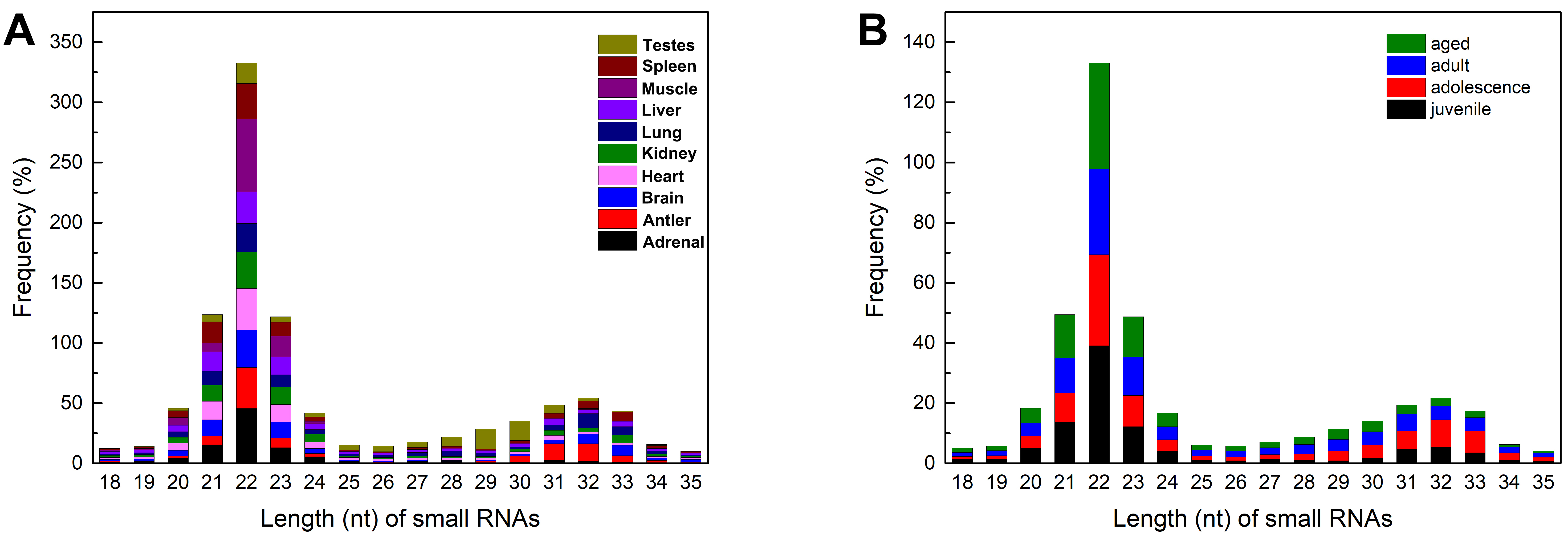

Supplement: Supplementary Figure 2 — Small RNA length distribution in the libraries. (A) The length distribution of unique small RNAs in the four developmental stages of Sika Deer. (B) The length distribution of unique small RNAs in ten organs of Sika Deer. [file Image_2.TIF]

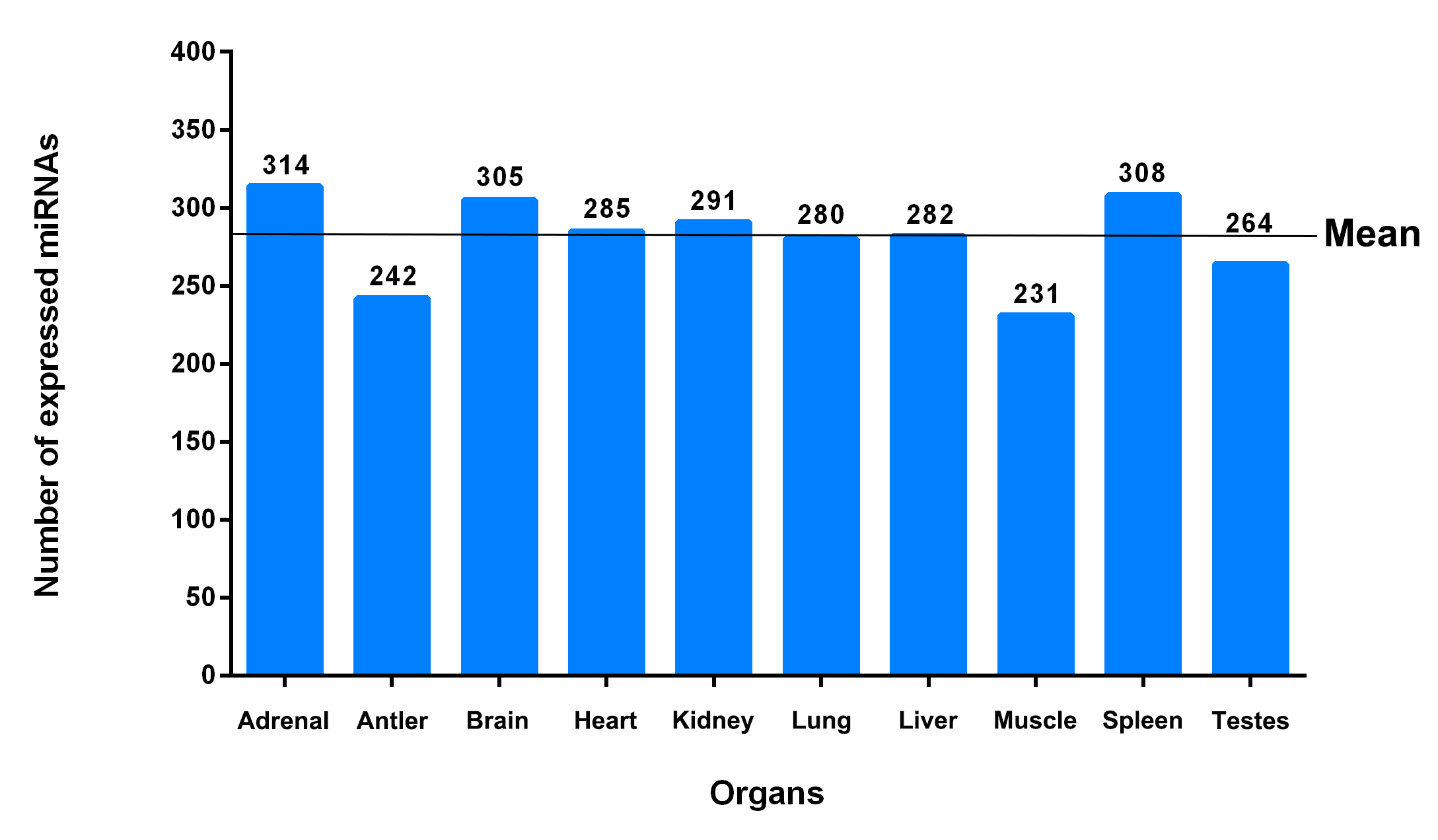

Supplement: Supplementary Figure 3 — miRNA expression in ten organs. [file Image_3.TIF]
